# Meckel’s diverticulum mimicking acute appendicitis in children: a retrospective cohort study

**DOI:** 10.1186/s12893-024-02323-4

**Published:** 2024-01-30

**Authors:** Ling Yan, Bi Zhou, Peng Wu, You Tian, Yi Lou

**Affiliations:** 1 Department of Pediatrics, Hangzhou Normal University, Hangzhou, China; 2 Department of Pediatrics, Suzhou Hospital of Anhui Medical University, Suzhou, Anhui China; 3 Department of Pediatric Surgery, Northwest Women and Children’s Hospital, Xi’an, Shaanxi China; 4 Department of Pediatric Surgery, Hangzhou Children’s Hospital, Hangzhou, 310014 Zhejiang China

**Keywords:** Meckel diverticulum, Appendicitis, Children, Abdominal pain, Heterotopic tissue

## Abstract

**Background:**

The aims of this study were to summarize the clinical presentation and histological results of 20 cases of complicated Meckel diverticulum (MD) who were presumed to have acute appendicitis before surgery, as well as to improve the diagnosis and treatment of complicated MD in children.

**Materials and methods:**

We retrospectively reviewed the records of 20 complicated MD admitted to our institution who were preoperatively diagnosed with acute appendicitis from January 2012 to January 2019. Patients were divided into the perforated MD group and the Meckel’s diverticulitis group. Patient demographics, clinical manifestations, laboratory data, auxiliary examinations, surgical methods, and the result of heterotopic tissue were recorded.

**Results:**

A total of 20 cases of complicated MD (perforated or diverticulitis) were identified. Children were aged from 3 to 13 years, with a mean age of 7.75 years (median 7.75; range, 1–13 years). Perforated Meckel’s diverticulum occurred in 5 of 20 (25%) cases. For perforated MD versus diverticulitis, no significant differences were found between age, time to intervention, length of hospital stay, and distance from the ileo-cecal valve. Heterotopic tissue was confirmed on histopathology in 75% of all patients, including 10 cases of gastric mucosa, 3 cases of coexistent gastric mucosa and pancreatic tissue, and 2 cases of pancreatic tissue. All patients underwent diverticulectomy or partial ileal resection under laparoscopy or laparotomy; two cases combined with appendectomy owing to slight inflammation of the appendix.

**Conclusions:**

The most common presentation of symptomatic MD is painless rectal bleeding; however, it can present symptoms of acute abdomen mimicking acute appendicitis. The key point of diverticulectomy is to remove the ectopic mucosa completely.

## Introduction

Meckel diverticulum (MD) is the most prevalent congenital anomaly of the gastrointestinal tract, occurring in 2–4% of the population [1, 2]. It is almost always located within 2 feet of the ileocaecal valve on the anti-mesenteric border at the terminal ileum. Histological MD often contains heterotopic mucosa, such as gastric mucosa, pancreatic tissues, and/or colonic mucosa, which can be seen in 50–60% of cases in surgical specimens [3]. Often asymptomatic, MD is commonly identified incidentally by abdominal exploration [4, 5]. When symptomatic, the most common clinical presentations were painless rectal bleeding, acute abdominal pain, small bowel obstruction, etc. [6]. . With its varied clinical manifestations for different individuals, it is difficult to prospectively diagnose Meckel’s diverticulum, especially in children [7].

Although perforation and diverticulitis are very rare complications in MD, Meckel diverticulum has been reported to mimic other acute abdominal pains like acute appendicitis, intestinal obstruction, pancreatitis, and so on [8, 9]. The aims of this study were to summarize the clinical manifestations, physical examinations, laboratory tests, and surgical outcomes of 20 cases of perforated Meckel’s diverticulum or Meckel’s diverticulitis who were presumed to have acute appendicitis before surgery.

## Materials and methods

With the approval of the institutional review board, we performed a retrospective review of 20 cases of complicated MD (perforated or diverticulitis) admitted to our institution who were preoperatively diagnosed with acute appendicitis from January 2012 to January 2019. Medical records were reviewed retrospectively, including clinical presentation at admission, laboratory values, intraoperative findings, surgical methods, and histological results. Only pediatric patients under 14 years old were included in this study. Patients were further divided into the perforated MD group and the Meckel’s diverticulitis group. The follow-up period ranged from 6 months to 5 years.

### Statistical analysis

SPSS Version 20.0 was applied for statistical analysis. All data were processed using descriptive statistical procedures for calculating means, standard deviations, frequencies, and percentages. The Kruskal–Wallis test was used to compare the perforated MD and the Meckel’s diverticulitis group. For categorical variables, Pearson’s chi square test was used to compare groups. *p* < 0.05 was considered to be statistically significant.

## Results

**Fig. 1:**
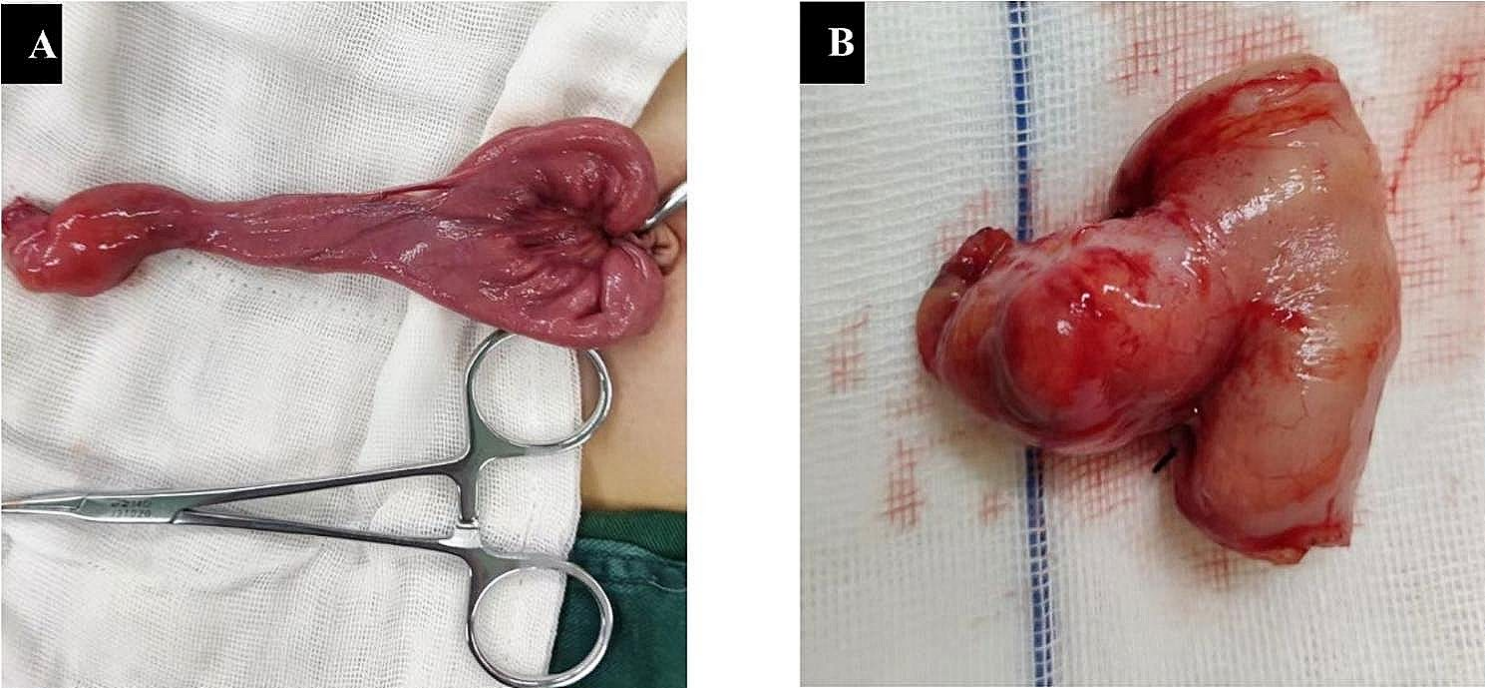
Appearance of Meckel’s diverticulum mimicking acute appendicitis. **A**: Meckel’s diverticulum was exteriorized through the umbilical wound in single incision laparoscopic surgery. **B**: Segmental bowel resection in a Meckel’s diverticulum

A total of 20 patients who underwent laparoscopy or laparotomy for MD mimicking acute appendicitis were identified (Fig. 1). There were 16 boys and 4 girls, with a male: female ratio of 4:1. Children were aged from 3 to 13 years, with a mean age of 7.75 years (median 7.75; range, 1–13 years). Perforated Meckel’s diverticulum occurred in 5 of 20 (25%) cases (Table 1). The main clinical symptoms were abdominal pain and/or nausea, vomiting, or fever. All patients (100%) had abdominal pain, and 14 (70%) had nausea or vomiting. Tenderness in the right lower quadrant was found in all patients, and 16 (80%) patients showed signs of peritonitis.

**Table 1:** Descriptive data for Meckel’s diverticulum(MD) mimicking acute appendicitis

Variable	Data
Gender, n (%)	
Male	16 (80%)
Female	4 (20%)
Intraoperative diagnosis	
Perforation	5 (25%)
Diverticulitis	15 (75%)
Clinical manifestations	
Abdominal pain	20 (100%)
Peritonitis	16(80%)
Nausea/Vomiting	14 (70%)
tenderness in the right lower	20 (100%)
Mean time to surgery [range], h	35.2 [10–72]
Auxiliary examinations, n (%)	
US	2 (10%)
CT	3 (50%)
Surgical methods	
Simple diverticulectomy	4 (20%)
Wedge-shaped excision	12 (60%)
Segmental bowel resection	4 (20%)

**Fig. 2:**
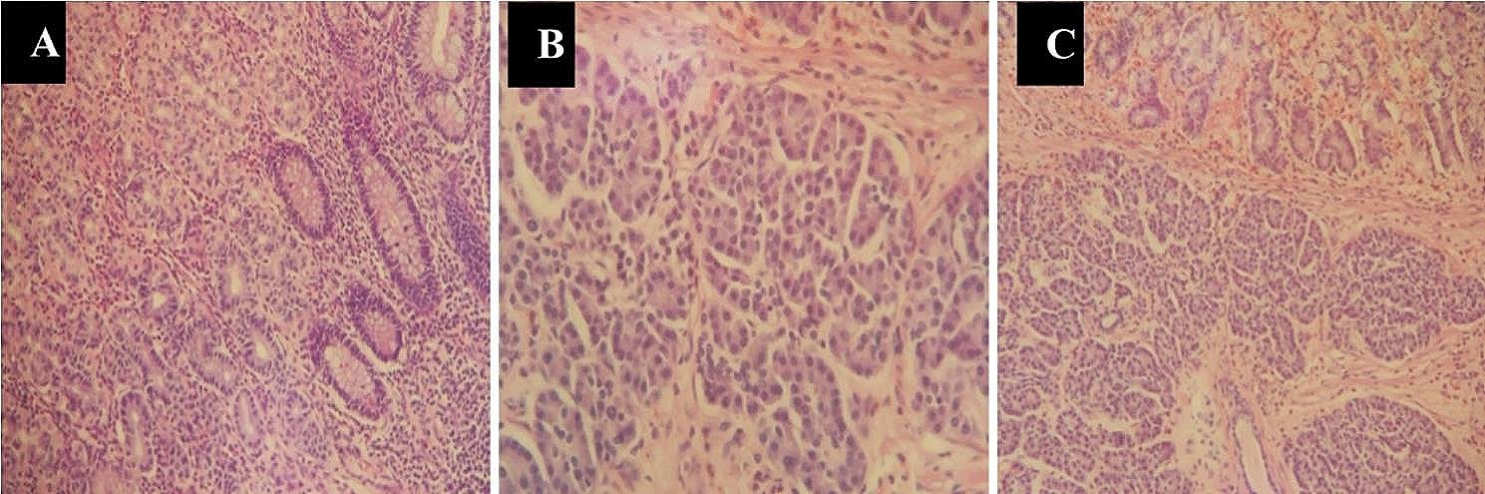
Postoperative histopathology revealed heterotopic gastricmucosa and/or ectopic pancreatic tissue in the diverticulum. **A**: Gastric heterotopia in a Meckel’s diverticulum. **B**: Ectopic pancreatic tissue in a Meckel’s diverticulum. **C**: Coexistence of heterotopic gastric mucosa and pancreatic tissue in a Meckel’s diverticulum

For perforated MD versus diverticulitis, no significant differences were found between age, time to intervention, length of hospital stay, and distance from the ileo-cecal valve. Only significantly higher serum levels of CRP before operation were found in the perforated MD group than diverticulitis group (Table 2). Ultrasonography (US) was performed in all patients, but only two patients offered a clue to MD (10%). Six patients underwent CT examination for further diagnosis of appendicitis, and three children showed a clue to MD (50%). Heterotopic tissue was confirmed on histopathology in 75% of all patients, including 10 cases of gastric mucosa, 3 cases of coexistent gastric mucosa and pancreatic tissue, and 2 cases of pancreatic tissue (Table 3; Fig. 2). In the perforated MD group, heterotopic tissue was found in all patients, including four cases of gastric mucosa and one case of coexistent gastric mucosa and pancreatic tissue. While in the diverticulitis group, heterotopic tissue was found in 66.7% of all patients, including 6 cases of gastric mucosa, 2 cases of coexistent gastric mucosa and pancreatic tissue, and 2 cases of pancreatic tissue.

**Table 2:** Comparison of continuous data for Meckel’s perforation and Meckel’s diverticulitis

Variable	Meckel’s Perforation	Meckel’s Diverticulitis	OR (95% CI)	*P* value
Median age [range], years	7.5 [3–11]	8 [3–13]	1.9 (-3.2–4.7)	0.809
Median WBC [range],nl	11 [8–24]	14 [6.7–25]	-2.6 (-3.7–7.4)	0.134
Median CRP [range],mg/l	99 [68–200]	27 [3–200]	37 (-140–15.8)	0.035
Median time to intervention [range], h	48 [24–72]	24 [10–72]	11.4 (-41–6.8)	0.799
Median Length of hospital stay [range], d	11 [8–13]	9 [3–12]	1.2 (-17.6–26.9)	0.586
Median distance from ileo-cecal valve [range], cm	30 [20–70]	40 [20–80]	10.6 (-4.8–0.21)	0.952

**Table 3:** Histological results of Meckel’s perforation and Meckel’s diverticulitis

Mucosal Type	Meckel’s Perforation	Meckel’s Diverticulitis	Total
Ectopic tissue	5	10	15
Gastric mucosa	4	6	10
Pancreatic tissue	0	2	2
Gastric and pancreatic	1	2	3

All patients underwent diverticulectomy or partial ileal resection under laparoscopy or laparotomy; two cases combined with appendectomy owing to slight inflammation of the appendix. Four children received a simple diverticulectomy. A wedge-shaped excision was performed in twelve patients, and four children underwent segmental bowel resection. All children recovered well with no anastomotic leak during follow-up, except for two cases of wound infection and two cases of intestinal obstruction, which occurred approximately two months after discharge and were cured after conservative treatment.

## Discussion

Failure to regress the omphalomesenteric duct during fetal life can result in various anomalies, including MD, fibrous band, patent vitelline duct, umbilical polyp, and umbilical cyst. Meckel diverticulum is the most frequent cause of gastrointestinal tract bleeding in children [10], and it is also one of the most prevalent congenital abnormalities in the gastrointestinal tract. Preoperative identification of symptomatic Meckel’s diverticulum is challenging, even though the majority of MD cases are asymptomatic [8]. The use of technetium-99 m (Tc-99 m) pertechnetate scintigraphy to detect ectopic gastric mucosa has been a well-established tool to diagnose MD in patients with repeated lower gastrointestinal bleeding or repeated attacks of intussusception, especially in older children or chronic intussusception, with relative high sensitivity and specificity [11, 12].

In our report, twenty children presented with acute abdomen (acute-onset abdominal pain, fever, and leukocytosis) preoperatively with the diagnosis of acute appendicitis, and none of them received Tc-99 m scanning. However, ultrasonography (US) was performed in all patients before surgery to diagnose appendicitis but is of limited value in diagnosing MD. Only two patients offered a clue to MD. Six patients underwent CT scans, and three children were shown a clue to MD. Most cases of MD occur before the age of 2 years in children, and between 25% and 50% of patients with clinical symptoms present younger than 10 years old [5]. In our study of twenty cases of MD mimicking acute appendicitis, the average age was 7.5 years old, which was older than the results of the previous studies. This discrepancy is most likely due to the fact that older children with acute abdominal pain are more likely to be diagnosed with appendicitis. Our study revealed that male patients were more likely than females to show symptomatic MD mimicking acute appendicitis with a male-to-female ratio of 4:1, which was similar to previous reports [7, 13]. The majority of children in our reported series presented with abdominal pain that was mostly localized in or around the right lower abdominal area.

MD perforation occurs in approximately 10% of the symptomatic children in the first year of life [2]. Diverticulum inflammation is usually accompanied by fever, nausea or vomiting, and abdominal pain, and these symptoms are often indistinguishable from acute appendicitis. Perforation of an MD will manifest with signs of diffuse peritonitis, usually localized in the lower abdomen [8]. In our series, 16 of the 20 patients showed signs of peritonitis; five children had perforated MD; and 15 patients had diverticulitis at laparotomy or laparoscopic surgery. The appendix was found to be normal in 18 cases; in 2 cases of perforated MD, the appendix showed slight inflammation. Ectopic tissue, which mainly consists of gastric mucosa and less commonly pancreatic tissue, jejunal tissue, or colonic tissue, is the cause of most complications of MD. It is estimated that the incidence of symptoms in MD with ectopic tissue can reach approximately 60%, while the incidence of symptoms in all MD is as low as approximately 2–4% [2, 8]. Symptomatic MD has a 10-fold increased incidence of heterotopic tissue [7, 14]. Chen et al. found that heterotopic tissue is the main cause of complicated diverticulum and suggested the removal of this tissue when it was incidentally found in pediatric patients [15]. The incidence of ectopic gastric mucosa in symptomatic MD is about 45–80% in pediatrics, according to the previous studies [7, 14, 16–18]. In this study, the incidence of ectopic mucosa in symptomatic MD patients was 75%, which was significantly higher than that in the incidentally found patients, especially in patients with perforated MD. The gastric mucosa or pancreatic tissue inside the diverticulum can secrete gastric acid or pancreatic juice, which may damage the diverticulum and neighboring intestine, thus leading to ulceration, bleeding, or inflammation.

The location of ectopic tissue in the diverticulum also affects the choice of operation methods [19]. In most cases, a simple diverticulectomy is sufficient when ectopic mucosae are located in the distal area of MD. However, in cases where broad-based diverticulum or ectopic tissues are found at the base of diverticulum, a wedge-shaped excision or segmental bowel resection is recommended instead [20]. In this study, a simple diverticulectomy was performed in only four children, wedge-shaped excisions were conducted in twelve patients, and four children received segmental bowel resection.

In conclusion, the preoperative diagnosis of MD remains a challenge for pediatricians and pediatric surgeons. Our study indicates that after acute appendicitis or other possible explanations have been ruled out, children with acute abdominal pain need to have a high index of suspicion for the diagnosis of complex MD. The key point of diverticulectomy is to remove the ectopic mucosa completely, and any other operative procedures should follow this principle.

## Data Availability

The dataset used and analyzed during the current study are available from the corresponding author on reasonable request.
